# Using the adherence‐efficacy relationship of emtricitabine and tenofovir disoproxil fumarate to calculate background hiv incidence: a secondary analysis of a randomized, controlled trial

**DOI:** 10.1002/jia2.25744

**Published:** 2021-05-21

**Authors:** David V Glidden, Moupali Das, David T Dunn, Ramin Ebrahimi, Yongwu Zhao, Oliver T Stirrup, Jared M Baeten, Peter L Anderson

**Affiliations:** ^1^ School of Medicine University of California San Francisco San Francisco CA USA; ^2^ Department of Epidemiology and Biostatistics San Francisco CA USA; ^3^ Gilead Sciences Inc. Foster City CA USA; ^4^ Centre for Clinical Research in Infection and Sexual Health Institute for Global Health University College London London UK; ^5^ University of Colorado Denver ‐ Anschutz Medical Campus Aurora CO USA

**Keywords:** counterfactual, PrEP, clinical trial design, tenofovir alafenamide, tenofovir disoproxil fumarate, Bayesian inference

## Abstract

**Introduction:**

Randomized trials of new agents for HIV pre‐exposure prophylaxis (PrEP) compare against emtricitabine and tenofovir disoproxil fumarate (F/TDF), without a placebo group. We used the well‐characterized adherence‐efficacy relationship for F/TDF to back‐calculate the (non‐PrEP) counterfactual background HIV incidence (bHIV) in a randomized trial of a novel PrEP agent and estimate comparative efficacy (to counterfactual bHIV).

**Methods:**

The DISCOVER trial (ClinicalTrials.gov: NCT02842086) randomized 5387 men who have sex with men (MSM) and transgender women who have sex with men and demonstrated non‐inferiority of emtricitabine and tenofovir alafenamide (F/TAF) to F/TDF (HIV incidence rate ratio [IRR] 0·47, 95% CI: 0·19 to 1.15). Tenofovir diphosphate (TFV‐DP) levels in dried blood spots (DBS) were assessed for all diagnosed with HIV and in a random 10% of the cohort. We used a Bayesian model with a diffuse prior distribution, derived from established data relating tenofovir diphosphate levels to HIV prevention efficacy. This prior, combined with the F/TDF seroconversion rate and tenofovir diphosphate levels in DISCOVER, yielded Bayesian inferences on the counterfactual bHIV.

**Results:**

There were six versus 11 postbaseline HIV infections (0.14 vs. 0.25/100 person‐years [PY]) on F/TAF and F/TDF respectively. Of the 11 on F/TDF, 10 had low, none had medium and one had high tenofovir diphosphate levels; among HIV‐negative controls, 5% of the person‐time years had low, 9% had medium and 86% had high TFV‐DP levels. A non‐informative prior distribution for counterfactual bHIV, combined with the prior for TFV‐DP level‐efficacy relationship, yielded a posterior counterfactual bHIV of 3·4 infections/100 PY (0.80 Bayesian credible interval [CrI] 1·9 to 5·9), which suggests a median HIV efficacy of 96% (0.95 CrI [88% to 99%]) for F/TAF and 93% (0.95 CrI [87% to 96%]) for F/TDF compared to bHIV.

**Conclusions:**

Based on the established connection of drug concentrations to PrEP prevention efficacy, a Bayesian framework can be used to estimate a synthetic non‐PrEP control group in randomized, active‐controlled PrEP trials that include a F/TDF‐comparator group.

## 1 INTRODUCTION

HIV pre‐exposure prophylaxis (PrEP) with oralemtricitabine and tenofovir disoproxil fumarate (F/TDF) is safe and highly effective for HIV prevention, when taken as directed in diverse at‐risk populations with rare seroconversions or drug resistance [1, 2, 3, 4, 5, 6, 7, 8, 9, 10, 11]. Where population‐level PrEP uptake is high, there have been promising declines in HIV incidence [6, 12, 13, 14, 15, 16, 17]. However, due to lower adoption and persistence on daily F/TDF in certain communities, particularly communities of colour and among transgender women, not all populations are benefiting equally from PrEP [18, 19]. There is a formidable pipeline of potent, safe antiretrovirals which could be used for PrEP and deserves urgent evaluation.

Given current multiple efficacious options for PrEP, designing clinical trials to evaluate the efficacy of novel PrEP methods has become increasingly challenging as a superiority design comparing a novel PrEP method to placebo is no longer feasible. Recent PrEP clinical trials have used an active‐controlled, non‐inferiority design comparing the novel PrEP product to F/TDF [20, 21]. The typical approach uses a narrow margin requiring a large number of incident infections. However, highly effective PrEP agents would result in low seroincidence in both the novel and comparator groups and thus few infections. This results in studies requiring many participants and long trial durations which delays evaluation and availability of new PrEP products.

Recognition of the limitation of requiring non‐inferiority trials for PrEP has led to the proposal of alternative clinical trial design, for example a design comparing HIV incidence on the new PrEP product with background HIV incidence (bHIV) in a population not on PrEP (herein referred to as the counterfactual bHIV) [22]. Ideally, this design would use more than one method to anchor the estimate of bHIV in the counterfactual non‐PrEP population. Possible approaches to estimating counterfactual bHIV include using routinely collected HIV surveillance data, recent clinical trial data which provide HIV incidence estimates, or correlating rectal gonorrhoea and HIV incidence [17, 23, 24, 25]. Glidden [26] reviews these methods, their comparative advantages, and how they might be used in PrEP regulatory approvals.

The present analysis proposes a novel method to explicitly estimate counterfactual bHIV using the well‐understood relationship between F/TDF adherence and PrEP efficacy, specifically objective adherence as measured by dried blood spots (DBS) among a group assigned F/TDF and observed HIV incidence in that group [1, 27]. By using observed HIV incidence during the trial in the F/TDF comparator group and understanding the adherence‐efficacy relationship for F/TDF, we can estimate what HIV incidence would have been if participants were not taking F/TDF for PrEP. We use data from the DISCOVER study, the first active‐controlled trial of a new PrEP product, F/TAF, with a F/TDF‐comparator group, to calculate counterfactual bHIV. Thus, we can estimate the efficacy of the new drug compared to a hypothetical placebo (or, no‐PrEP) group, providing an interpretable efficacy estimate for a new PrEP drug.

## 2 METHODS

### 2.1 The DISCOVER trial

The DISCOVER trial is a randomized, double‐blind, multicentre, active‐controlled non‐inferiority trial following HIV‐negative adult cisgender men who have sex with men (MSM) and transgender women who have sex with men at high risk of HIV acquisition; primary results have been previously reported [20]. Individuals were randomized 1:1 to daily blinded tablets of coformulated F/TAF (200/25 mg) or coformulated F/TDF (200/300 mg). At each visit, all individuals had clinical assessments, were tested for HIV, screened for sexually transmitted infections (STIs), and had DBS collected to monitor adherence. In persons with incident HIV infection and in a randomly pre‐selected subset of 10% of all participants (a case‐cohort design) [28], DBS samples were tested for tenofovir diphosphate (TFV‐DP) levels in red blood cells [29]. TFV‐DP levels were categorized in three adherence categories: <350 fmol/punch (<2 tablets/week), 350 to <700 fmol/punch (2 to 3 tablets/week) and ≥700 fmol/punch (≥4 tablets/week). Methods and benchmarks for F/TAF and F/TDF have been previously described [30, 31]. Data were collected between 13 September 2016 and 22 February 2019. This study was undertaken in accordance with the Declaration of Helsinki and were approved by central or site‐specific review boards or ethics committees. All participants provided written informed consent.

### 2.2 Statistical methods

Ancillary data observed in DISCOVER, including STIs, peri‐enrolment HIV infections and drug levels in the F/TAF and F/TDF arms, suggest substantial reduction in HIV incidence in both arms compared to counterfactual bHIV. Bayesian analysis incorporated these data with prior studies to calculate background HIV incidence in the counterfactual population of DISCOVER participants had they not been adherent to F/TAF or F/TDF and the calculated the overall HIV prevention efficacy by study arm. Our analysis was based on aggregate data from DISCOVER with data on the follow‐up and HIV infections by study arm and adherence category.

Our model for HIV incidence is presented in the supplement. We then applied Bayesian statistics (methods for formally combining observed data with external information – both prior belief and results from previous studies [32]) to give us a framework to import information about F/TDF pharmacology to estimates bHIV and F/TAF and F/TDF efficacy without complex mathematical approximations. The method combines a Poisson likelihood for observed data, adjusted for case cohort sampling [33], with prior distributions.

Our method combined observed HIV incidence, drug levels on F/TDF and prior assumptions on the relationship between adherence and HIV protection to inform an estimate of the bHIV [2]. The prior was developed from a study of F/TDF, in a population of MSM and transgender women, by pooling data from iPrEx randomized and open‐label extension (OLE) phases [1, 34]. We modelled the relationship between seroconversion with a TFV‐DP DBS level as $\beta_{l}$ = $\beta_{0}+\beta_{1}l$ where l=0 if x<350, l=1 if x∈[350,700) and l=2 if x≥700 fmol per punch, which fit similarly to a spline model with continuous TFV‐DP levels (p = 0.10). Based on this, we estimated the HIV prevention efficacy associated with TFV‐DP levels as 350 (low), 350 to <700 (medium) and ≥700 (high) fmol/punch were assumed to provide 0%, 86% and 98% HIV protection respectively (Table 1).

**Table 1:** iPrEx OLE and DISCOVER results by tenofovir drug levels

TFV‐DP in DBS, fmol/punches	(A)			(B)	
	iPrEx OLE^2^			F/TDF arm of Discover	
	Average adherence^a^	Relative risks vs. placebo (95% CI)	HIV prevention efficacy, %	Acquired infections, n	Estimated PY in case cohort (%)^b^
<350	Low: <2 tablets/wk	1.19 (0.76, 1.87)	0	10	219 (5)
350–<700	Moderate: 2 to 3 tablets/wk	0.14 (0.02, 0.76)	86	0	395 (9)
≥700	High: ≥4 tablets/week	0.02 (0.00, 0.49)	98	1	3772 (86)

CI, confidence interval; DBS, dried blood spots; F/TDF, co‐formulated emtricitabine and tenofovir disoproxil fumarate; OLE, open‐label extension; PY, person years; TFV‐DP: tenofovir diphosphate; wk, week.

^a^ Over previous month

^b^ estimated from design of case‐cohort study and Bayesian model.

Five participants had suspected peri‐enrolment infections between testing HIV negative at their screening visit and acquiring HIV by week 4 [20]. This could inform an estimate of $\lambda_{0}$. Assuming a 14‐day average lag‐time between infection and a positive test, we estimated the infections were observed over approximately 173 person‐years (PY) of possible follow‐up, resulting in a background HIV incidence rate (IRR) of 2.9/100 PY (0.95 CI: 0.9 to 6.7) in the screened/enrolled participant population.

For bHIV, we used two specifications: a (i) flat prior and (ii) sceptical or conservative prior. The flat prior had minimal impact on the bHIV estimate and approximates conclusions of non‐Bayesian methods. The flat prior, combined with the seroconversion rate of F/TDF and TFV‐DP levels, permitted Bayesian inferences on the estimate of counterfactual bHIV. Sensitivity analyses are presented in the supplement.

Analyses used STAN [34] implemented in R [35] to sample 20,000 realizations from posterior distributions. This produced sampled values from the posterior distribution for HIV incidence on F/TAF, F/TDF and the bHIV. For each sample, estimates of posterior efficacy, averted infections (and the number of people needed on either drug to prevent one new HIV infection) were calculated. These yielded samples from the posteriors for these estimands (see supplement). We summarized bHIV distribution using posterior medians and 80% posterior credible intervals (CrI). These CrIs are an analogue to the confidence interval and represent the range of posterior belief in the value of parameters consistent with observed data under the model and prior assumptions. For efficacy estimates, we used the 95% posterior density CrI. Further details are provided in the supplementary materials.

## 3 RESULTS

### 3.1 Summary of key DISCOVER trial results

The DISCOVER trial enrolled and randomized 5387 participants (2694 to F/TAF, 2693 to F/TDF) with 8756 PY of follow‐up at the primary endpoint [20]. The primary analysis included 22 participants who were diagnosed with HIV. Of the 22, one on F/TAF and four on F/TDF were suspected to have acquired HIV infection prior to baseline based on the totality of available evidence, including exposure history, timing of HIV testing and genotypes. The 17 post‐baseline acquisitions are the focus of this analysis: six for F/TAF (HIV incidence rate: 0.14/100 PY [95% CI 0.05 to 0.30]) and 11 for F/TDF (HIV incidence rate: 0.25/100 PY [95% CI 0.13 to 0.45]). HIV incidence was low in both the F/TAF and F/TDF groups in the setting of high STI incidence in both groups (pooled rates of 21.0/ and 9.9/100 PY for rectal gonorrhoea and syphilis respectively). As previously reported, adherence by self‐report, pill count and the objective measure of TFV‐DP in red blood cell DBS was very high in both groups in the study.

### 3.2 Distribution of HIV infections and person‐Time in the F/TDF arm by TFV‐DP DBS adherence category

Data from iPrEx OLE established the relationship between TFV‐DP in DBS and average adherence in tablets/week (low, moderate, high) and HIV prevention efficacy (0, 86% and 98% respectively) (Table 1A). We report the number of HIV infections and the estimated person‐years in the F/TDF arm in each of these adherence categories (Table 1B). High adherence was observed in most of the person‐years in the DISCOVER F/TDF arm (86%), with only one infection in this category. Low adherence was observed in only 5% of the person‐time; however, 91% of HIV infections occurred in this category. There were no infections among the 9% of person‐years with moderate adherence.

### 3.3 Bayesian estimates of posterior efficacy distributions for F/TAF and F/TDF

**Figure 1:**
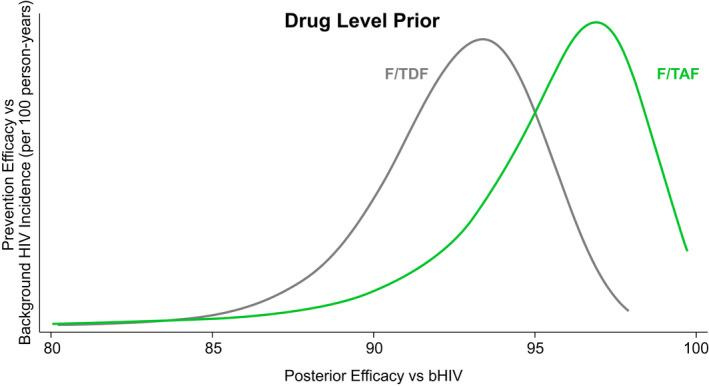
Bayesian estimates of posterior efficacy distributions for F/TAF and F/TDF. F/TAF, co‐formulated emtricitabine and tenofovir alafenamide; F/TDF, co‐formulated emtricitabine and tenofovir disoproxil fumarate. F/TAF, co‐formulated emtricitabine and tenofovir alafenamide; F/TDF, co‐formulated emtricitabine and tenofovir disoproxil fumarate.

**Figure 2:**
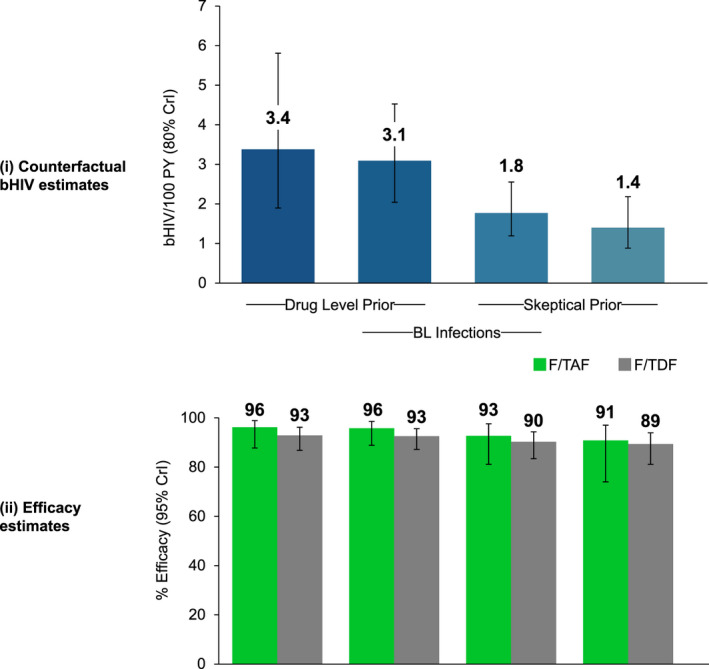
Estimates of counterfactual bHIV by the adherence‐efficacy method under varying assumptions and corresponding efficacy estimates.

Using a prior distribution based solely on TFV‐DP levels, the Bayesian model yielded a median posterior bHIV of 3.4 infections/100 PY (80% CrI 1.9 to 5.9) (Figure 2i, column A), that is counterfactual HIV incidence in the absence of PrEP would have been 3.4 infections/100 PY. Given that observed HIV incidence was 0.14 infections/100 PY (95% CI 0.05 to 0.30) for F/TAF and 0.25 infections/100 PY (95% CI 0.13 to 0.45) for F/TDF, we calculated the posterior densities for HIV prevention efficacy in each group (Figure 1). Using the flat prior based only on adherence by TFV‐DP in DBS, median HIV incidence reduction in the F/TAF and F/TDF arms was 96% (0.95 CrI 88% to 99%) and 93% (0.95 CrI 87% to 96%) respectively (Figure 2ii, column A).

### 3.4 Estimates of the DISCOVER counterfactual bHIV by adherence‐efficacy method under varying assumptions

In a series of sensitivity analyses, we incorporated additional information to the prior based on TFV‐DP levels alone. As increasingly conservative information was added, estimates of counterfactual bHIV declined. Counterfactual bHIV estimates shown in Figure 2i, and Figure 2ii summarizes the corresponding posterior HIV prevention efficacy estimates for F/TAF and F/TDF.

The first sensitivity analysis incorporated the estimate of HIV incidence based on suspected baseline HIV infections. As described above, five participants had peri‐enrolment HIV infections. If we assume that pre‐enrolment incidence reflects bHIV, this information can be used in the Bayesian estimation by incorporating them into the likelihood (see supplement). Incorporating data from baseline infections shifted the bHIV estimate lower to 3.1 infections/100 PY (80% CrI 2.0 to 45) (Figure 2i, column B); however, efficacy estimates remained the same at 96% (95% CrI 89% to 99%) for F/TAF and 93% (95% CrI 87% to 96%) for F/TDF (Figure 2ii, column B). Next, we considered a sceptical prior by incorporating a conservative prior belief about bHIV by making a subjective assumption that bHIV was low (e.g. assuming that low HIV incidence observed in DISCOVER was due to low background HIV incidence in the participant population rather high efficacy of either drug). We specified a prior for bHIV with median 0.50 infections/100 PY, with 0.80 probability that the incidence lay between 0.26 and 0.95 infections/100 PY. Incorporating this assumption pulled the posterior for bHIV, and thus the preventive efficacy, estimates lower. With these assumptions, we estimated a counterfactual bHIV of 1.8 infections/100 PY (80% CrI 1.2 to 2.6) (Figure 2i, column C), with efficacy estimates of 93% (95% CrI 81% to 98%) for F/TAF and 90% (95% CrI 83% to 94%) for F/TDF (Figure 2ii, column C). When considering only the sceptical prior by removing the baseline infection data, the estimate for counterfactual bHIV was 1.4 infections/100 PY (80% CrI 0.9 to 2·2) (Figure 2i, column D), with efficacy estimate of 91% (95% CrI 74% to 97%) for F/TAF and 89% (95% CrI 81% to 94%) for F/TDF (Figure 2ii, column D).

The results are also dependent on drug levels among seroconverters in DISCOVER. For instance if the person with high adherence was moved to the lowest adherence category, then the bHIV median would have changed from 3.4 to 3.8/100 PY. However, if all 11 F/TDF infections in DISCOVER occurred in the high adherence group and if we used that prior, then the median bHIV would have been estimated to be 0.20/100 PY, with been little evidence for efficacy of F/TAF or F/TDF.

We explored the role of confounding between study drug adherence behaviour and sexual risk behaviour by assuming unequal baseline risk across categories of adherence using the model described in Volk et al. [6] With a 9‐fold higher risk among the lowest adherence category compared to the highest (ϕ=3), the bHIV posterior median was 1.1 (80% CrI 0.7 to 1.7) (80% CrI 0.7 to 1.7) (Figure S1, column E), with median HIV efficacy estimates of 88% (95% CrI 67% to 96%) and 71% (95% CrI 51% to 83%) for F/TAF and F/TDF respectively (Figure 2iI, column E). Figure S1 (columns F to H) provide counterfactual bHIV estimates and corresponding median efficacy estimates that assessed this potential confounding along with other sensitivity assumptions. As estimates of counterfactual bHIV decreased, the differential between efficacy estimates for F/TAF and F/TDF increased. A background incidence of 3.1/100 PY would translate to efficacies of 100*(1 to 0.14/3.1) = 95% and 100*(1 to 0.25/3.1) = 92%, for F/TAF and F/TDF respectively. But, 0.3/100 PY would translate to efficacies of 100*(1 to 0.14/0.30) = 53% and 100*(1 to 0.25/0.30) = 16%, for F/TAF and F/TDF respectively.

If the direction of the confounding was reversed, for example, higher sexual risk behaviours were associated with higher adherence as found in some contexts [27, 36], the posterior median bHIV was 6.3 per 100 person years (0.95 CrI 3.5 to 10.9), leading to >99% efficacy in both arms.

### 3.5 Averted infections and number needed to prevent

Using the estimates for counterfactual bHIV, we calculated both the (1) number of infections averted by either study drug and (2) number of individuals who would need to be treated to prevent one HIV infection (i.e. number needed to prevent) (Figure 2iii).

## 4 DISCUSSION

The strongest proof‐of‐concept for a PrEP agent comes from evidence of prevented infections [26, 37]. For the first studies of F/TDF for PrEP, that evidence was obtained from direct comparison to placebo; however, placebo‐controlled PrEP trials are no longer feasible, given demonstration of efficacy of F/TDF and global recommendations for its use. Evaluating efficacy of new drugs for HIV prevention by using an active‐controlled, non‐inferiority trial design have become even more challenging in recent years due to the high efficacy of other recently developed HIV prevention drugs [20, 22]. Evidence for new PrEP agents tested in new trials will require triangulation of evidence of plausibly high background incidence (by baseline, screening infections, HIV transmitting sexual practices, STIs); effective product use (by pharmacology or directly observed administration); and low observed HIV incidence on the investigational product. We formally incorporated external data collected by a trial (the former two elements) and well as HIV seroconversion post‐randomization. This approach may permit more efficient inference and possibly smaller trials. Using this method, one is able to estimate a counterfactual bHIV and essentially add a third placebo arm to a trial evaluating a new drug and comparing it to F/TDF for HIV PrEP.

We present an approach using the well‐understood relationship between adherence and HIV prevention efficacy with F/TDF gained from experience of prior trials with objective adherence data from TFV‐DP in DBS to calculate a counterfactual bHIV in the trial participants. TFV‐DP in DBS is a unique adherence assessment given its 2.5‐week half‐life, so levels represent cumulative adherence in the preceding six to eight weeks [30, 31].

This technique could potentially be used with F/TAF; however, as only six HIV infections from DISCOVER on F/TAF, additional data would be needed to fully develop this relationship. This technique could be adapted to use in studies of long‐acting agents for the prevention of other infectious diseases using data regarding time to injection and/or drug levels, and potentially adapted for preventative vaccines if suitable immune correlates are identified [38].

By utilizing the well‐characterized adherence‐efficacy relationship for F/TDF, we were able to estimate the following: (1) counterfactual background HIV incidence in the for F/TDF arm of the study; (2) high efficacy of both study drugs based on the estimate of bHIV; (3) estimated number of HIV infections averted by each study drug; and (4) number needed to receive PrEP to prevent one new HIV infection for each study drug.

We used a variety of deliberately conservative assumptions and interrogated a wide range of scenarios. We performed an analysis which assumed that the ratio of 9‐fold reduction in HIV risk from lowest to highest adherence categories; thus we have extended this approach to allow for sensitivity analysis to unmeasured confounding, which can be a threat to validity. Importantly, results were not sensitive to even a large degree of confounding. Exploring confounding (assuming those with low adherence had 9‐fold higher risk behaviour) added to each of the models, suggesting that F/TAF may have higher efficacy than for F/TDF, with lower bHIV incidence. Notably, in some studies adherence was associated with a risk of high sexual practices [27, 36] – and we also explored this possibility, which led to the highest estimate of bHIV (6.3/100 PY) and efficacy of 99% for both F/TAF and F/TDF.

**Figure 3:**
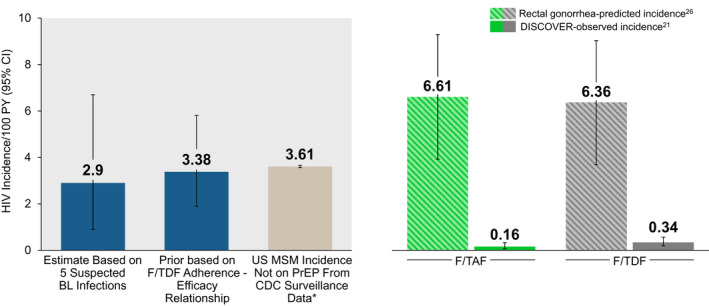
Comparing Counterfactual Estimates of Background Placebo HIV Incidence. *Based on Centers for Disease Control and Prevention (CDC) surveillance data [42]. BL, baseline; F/TAF, co‐formulated emtricitabine and tenofovir alafenamide; F/TDF, co‐formulated emtricitabine and tenofovir disoproxil fumarate.

Alternative methods for evaluating the counterfactual bHIV in clinical trials for PrEP, including use of other pharmacology data [39, 40, 41], using the observed correlation between rectal gonorrhoea and HIV incidence [25, 26], HIV surveillance data [35], run‐in periods [26], or by using the acute HIV infection or recency assays the screened population all have their benefits and limitations [43]. The historic correlation between rectal gonorrhoea and HIV incidence may be an overestimate of bHIV in the current area with greater rates of viral suppression and PrEP uptake (DISCOVER bHIV estimates >6/100 PY, Figure 3). Using surveillance data to estimate counterfactual bHIV is only possible in locations where the data are collected and have stable HIV testing rates; additionally, clincial trial participants may not be similar to the surveillance dataset. Likewise recency assay‐based estimates depend on the performance characteristics of the particular assay chosen, understanding of the HIV incidence and prevalence, gender distribution and proportion on HIV therapy and virally suppressed in the population to be studied [27, 40]. The benefit of our proposed method is that it is unaffected by the above considerations and provides a direct, post‐randomization, concurrent estimation of ongoing HIV incidence that would have occurred if the F/TDF participants were not on PrEP during the trial. Our estimate of counterfactual bHIV (3.4 infections/100 PY) by the Bayesian method is consistent with the DISCOVER estimate using baseline HIV infection incidence (2.9 infections/100 PY) and estimated HIV incidence using CDC surveillance data for new diagnoses in MSM not on PrEP (3.6 infections/100 PY) (Figure 3). Moreover, the Bayesian framework could be adapted to any of the alternate counterfactual estimation methods or any combination of them.

Limitations of our method include its highly parametric formulation which may fit data imperfectly. The bHIV estimate relies of HIV infections on F/TDF for its calculation: the lower the number of HIV infections, the more imprecise the estimate of bHIV. The TFV‐DP in DBS adherence‐efficacy relationship is best understood in MSM. However, plasma tenofovir has been correlated with protection among heterosexual men and women in Partners PrEP [41] so plasma tenofovir adherence‐efficacy could be used. Lastly, any drug level is a surrogate measured at a specific visit and not necessarily at the time of an HIV exposure; hence it may imperfectly capture the key level which determines protection. Despite these limitations, the Bayesian framework using the well‐characterized adherence‐efficacy to calculate bHIV in the F/TDF control arm of a PrEP trial offers a promising option for estimating a concurrent counterfactual bHIV estimate.

## 5 CONCLUSIONS

The field of HIV prevention is at a cross roads. New prevention options are necessary as not all those at risk for HIV are benefiting from currently available PrEP. The methods described in the present analysis allow for a reasonable estimation for counterfactual bHIV incidence in clinical trials for new PrEP drugs which include F/TDF as an active control. This is of importance as new HIV prevention trials cannot include a placebo arm for feasible reasons. Work is ongoing to develop power calculations based on this strategy, which may reduce the sample size required for future PrEP trials [44]. In combination, the approach outlined may accelerate the understanding of the efficacy of new HIV prevention drugs and help increase options for individuals at risk for HIV and uptake of PrEP to meet our shared goals of ending the epidemic.

## COMPETING INTEREST

DVG reports accepting fees from Gilead Sciences and Merck. DTD has received personal fees from Gilead Sciences. MD, RE, YZ and JMB are employees of Gilead and shareholders of Gilead stock. PLA reports grants and personal fees from Gilead Sciences during the conduct of the study, and reports grants from Gilead Sciences outside of the submitted work. OTS reports no conflicts of interest.

## AUTHORS’ CONTRIBUTIONS

RE and YZ analysed the clinical data, which were reviewed and interpreted by MD. PLA analysed the TFV‐DP samples in DBS. DVG and OTS performed the statistical modelling for the current analysis, and results were reviewed and interpreted by DVG, MD, OTS, JMB and PLA. The first draft was written by DVG and MD. All authors contributed to edits of the final manuscript and made the decision to submit the manuscript for publication. All authors had access to the data and are responsible for data integrity and completeness.

## Supporting information


**Table S1**. Estimates and Interval Estimates For The Frequentist And Bayesian Estimator Of The Rate of HIV on the F/TDF arm
**Figure S1**. Estimates of counterfactual bHIV by the adherence‐efficacy method under varying assumptions and corresponding efficacy estimates and quantitative bias analysis
