# Supplementary material for: Using the adherence‐efficacy relationship of emtricitabine and tenofovir disoproxil fumarate to calculate background hiv incidence: a secondary analysis of a randomized, controlled trial
Source: J Int AIDS Soc. 2021 May 21;24(5):e25744. doi: 10.1002/jia2.25744 (PMC8140182; doi:10.1002/jia2.25744)
Supplement: Supplementary file 1 — Table S1. Estimates and Interval Estimates For The Frequentist And Bayesian Estimator Of The Rate of HIV on the F/TDF arm Figure S1. Estimates of counterfactual bHIV by the adherence‐efficacy method under varying assumptions and corresponding efficacy estimates and quantitative bias analysis [file JIA2-24-e25744-s001.docx]

## Statistical Methods and Formulae

*Model for HIV Incidence*

The number of HIV infections on the F/TAF arm was modeled as Poisson with rate $\lambda_{1}$ and likelihood

$L_{1}(\lambda_{1})=\lambda_{1}^{\Delta_{1}}exp(-\lambda_{1}T_{1})$ (1)

where $(\Delta_{1},T_{1})$ were the number of HIV infection and total PY of follow-up on F/TAF, respectively. Let $\lambda_{0}$ denote the bHIV rate in the sample. If we have TFV-DP levels available on F/TDF, we represent the rate of HIV in TFV-DP stratum $l$ as

$\lambda_{2l}=exp\{log(\lambda_{0})+\beta_{l}\}$ (2)

where $(l=0,1,2)$, are the three TFV-DP drug categories. For those selected for the case cohort sampling on the F/TDF arm, the likelihood, $L_{2c}(\lambda_{0},\beta,\rho)$ was proportional to

$\underset{l}{\prod}\lambda_{2l}^{\Delta_{cl}}exp(-\lambda_{2l}T_{cl})\rho_{l}^{T_{cl}}$ (3)

where $\Delta_{cl}$ is the number of HIV infections, $T_{cl}$ is and total follow-up time (in the case-cohort sample), and $\rho_{l}$, was the fraction of follow-up time on the F/TDF arm with drug level $l$, $(l=0,1,2)$. For those randomized for F/TDF and not selected for the case/cohort sample, the likelihood was

$L_{2\overline{c}}=\overset{2}{\underset{l=0}{\sum}}\rho_{l}exp(-\lambda_{2l}T_{\overline{c}})$ (4)

where $T_{\overline{c}}$ is the total follow-up time among those without drug levels measured (neither cases nor among the cohort). Let ${T_{2}= T}_{\overline{c}}+T_{c1 +}T_{c2 +}T_{c3}$.

If we had follow-up in a non-PrEP period, during which $\Delta_{0}$ HIV infections were observed during a follow-up period $T_{0}$ , this would give the likelihood contribution

$L_{0}=\lambda_{0}^{\Delta_{0}}exp(-\lambda_{0}T_{0})$ (5)

which can be extended to allow for unmeasured confounding by allowing for varying rate heterogeneity across (latent) F/TDF adherence categories $(l=0,1,2)$,

$L_{0}=\sum_{l=0}^{2}\rho_{l}exp(-\lambda_{0l}T_{0})=\sum_{l=0}^{2}\rho_{l}exp(-\phi^{(1-l)}\lambda_{01}T_{0})$, (6)

where, $\lambda_{00}/\lambda_{01}=\phi$ and $\lambda_{01}/\lambda_{02}=\phi$ with $\phi\geq1$. When $\phi=1$, the risk is unconfounded by the (latent) adherence categories. However, if $\phi=2$, then the lowest adherence group has twice the bHIV rate of the medium adherence group which has a bHIV rate twice that of the high adherence group.

Thus, this in the absence of PrEP, this would allow for seroconversion on the F/TDF to be associated with drug levels, by some magnitude beyond any effect of TFV-DP on protection. The ratio is given by

$\lambda_{22}/\lambda_{20}=\frac{\phi exp\{log(\lambda_{01})+\beta_{2}\}}{\phi^{-1}exp\{log(\lambda_{01})+\beta_{0}\}}=\phi^{2}exp\{\beta_{2}-\beta_{0}\}$

Note, the ratio of the risk on F/TDF in the low adherence group compared to the high adherence group would be discounted by $\phi^{2}$ due to potential confounding. The data likelihood for the trial is the product of $L_{1}(\lambda_{1})$, $L_{2c}(\lambda_{0},\beta,\rho)$, $L_{2\overline{c}}(\lambda_{0},\beta,\rho)$ and $L_{0}(\lambda_{0})$.

*Construction of Priors*

For F/TAF incidence, we employed a flat prior for $f(\lambda_{1})$. Such a prior will impose minimal assumptions and approximate frequentist inference. The purpose of our method is to combine the observed HIV incidence and drug levels on the F/TDF with prior assumptions on the relationship between adherence and HIV protection to inform (2). The prior modeled the relationship between seroconversion with a FTP-TP DBS level as $\beta_{l}$ = $\beta_{0}+\beta_{1}l$ where $l=0$ if $x<350$, $l=1$ if $x\in[350,700)$ and $l=2$ if $x\geq$700 fmol/punch. We fit this model to the OLE data and then used the parameter estimates and standard errors obtained to inform a bivariate normal distribution for the relevant prior in our Bayesian analysis of the DISCOVER data. A key assumption here is the implicit causality of drug exposure on HIV protection due to PrEP. Causality is supported by the fact that mechanistically the delivery of the drug is mechanism of the drug supporting it as being a part of the causal pathway. Implicitly, there is an assumption of no unmeasured confounders between the drug level and the exposure. This assumption cannot be fully verified. The parameter estimates were $\mu=$(0.18,-2.14) with estimated variance covariance matrix. The parameter estimates were $\mu=$(0.18, -2.14) with estimated variance covariance matrix

$$\Sigma=\left[ \begin{matrix} 0.053 & -0.028 \\ -0.028 & 0.765 \end{matrix} \right]$$

The value of $\mu$[1] = 0.18 tends to support causality because it implies that HIV risk in those who were randomized to PrEP but did not take it have similar HIV risk to those in placebo. The informative prior distribution, $f(\beta)$ ,combined with the data on HIV and drug levels on the F/TDF arm provided the posterior distribution for bHIV, as multivariate normal $(\mu,\Sigma)$. We employed a flat prior for $\{\rho_{0},\rho_{1},\rho_{2}\}$.

For bHIV incidence we used two strategies a (i) flat prior as well as (ii) a conservative prior with log-normal distribution Normal (-5.3,0.5). Because of concerns about flat priors, we also explored a variety of non-informative and weakly informative priors which produced very similar results. We performed the following sensitivity analyses: (1) drug level prior informed by incorporating suspected baseline (BL) HIV infections; (2) skeptical prior incorporating a low estimate of bHIV in DISCOVER, a conservative assumption to address concerns that DISCOVER had a low bHIV due to the recruitment of low-risk individuals instead of high efficacy of both drugs; and (3) a quantitative bias analysis, which assessed potential confounding between study drug adherence and risk behavior. The skeptical prior for bHIV had a median incidence of 0.50/100 PY, with 0.80 probability that the incidence lay between 0.26/ and 0.95/100 PY. This background HIV incidence was lower than that observed in the placebo arm of any randomized PrEP trial to date and not consistent with HIV surveillance data for many of the sites in DISCOVER,[42] serving as a strongly skeptical benchmark.

For (3) above, if participants more adherent to study medication also tended to be at lower risk of HIV infection, then the back-calculation method could be biased because it does not allow for confounding between adherence and risk of HIV infection. We induced unobserved confounding by assuming that there was unequal baseline risk across the categories of adherence. The magnitude of confounding was represented by a parameter $\phi$, which assumed that the degree of risk from the low vs. high adherence group is $\phi$^2^ greater. We used $\phi=3$, which assumed that participants with low adherence had 9-fold higher risk behavior.

*Posterior Estimands*

The F/TDF arm is modeled as a mixture of Poisson distributions. Such a model is not, itself, Poisson and thus lacks a constant hazard. However, in settings where baseline hazards are less than 10 to 20/100 PY, it will be approximately constant. An expression for the hazard of the Poisson mixture is

$$\lambda_{2}(t)=\frac{\sum_{l}\rho_{l}\{1-exp(-\lambda_{2l}t)\}}{\sum_{l}\lambda_{2l}^{-1}\rho_{l}\{1-exp(-\lambda_{2l}t)\}}$$

here we choose the least false value of the hazard as its value at the average follow-up $\overline{\lambda}_{2}=\lambda_{2}(\overline{\tau})$. We examined simulation in a variety of mixed Poisson settings and found that $\overline{\lambda}_{2}$ well approximated the result of fitting a homogenous Poisson to the same data. This value of the estimate is further reinforced by the posterior means of the overall rate on the TDF arm allowing for heterogeneity (when a non-informative prior is used) being similar to the frequentist estimate. This can be seen in Table S1. We drew samples of

$\Theta_{1}=100(1-\lambda_{1}/\lambda_{0})$, the preventive efficacy of F/TAF in the population compared to bHIV rate

$\Theta_{2}=100\{1-[\sum_{l}\rho_{l}\lambda_{2l}exp(-\lambda_{2l}\tau)]/[\sum_{l}\rho_{l}\lambda_{2l}\lambda_{0}]\}$, the preventive efficacy of F/TDF compared to bHIV where $\tau$ is the average follow-up on the F/TDF arm.

The number of averted infections on F/TAF and F/TDF was estimated by and , respectively where T = ($T_{1}+T_{2})/2$, the average total follow-up per arm.

$\lambda_{0}$: the bHIV rate from the posterior distribution

## Table S1: Estimates and Interval Estimates for the Frequentist and Bayesian Estimator of the Rate of HIV on the F/TDF arm.

|  | F/TDF rate* | F/TDF 95% CI^¥^ | |
| --- | --- | --- | --- |
|  |  | **Lower** | **Upper** |
| Frequentist Estimate | 0.25 | 0.13 | 0.45 |
| Flat Prior, No Baseline Infections | 0.25 | 0.13 | 0.42 |
| Flat Prior, Baseline Infection | 0.24 | 0.13 | 0.37 |
| Flat Prior, No Baseline Infections, $\phi=3$ | 0.23 | 0.13 | 0.38 |
| Flat Prior, Baseline Infection, $\phi=3$ | 0.26 | 0.18 | 0.49 |

CI, confidence interval; F/TDF, co-formulated emtricitabine and tenofovir disoproxil fumarate

* Maximum likelihood estimator for frequentist case, posterior mean of $\overline{\lambda}_{2}$ in the Bayesian setting

^¥^ 95% confidence interval for frequentist case. 95% credible interval for Bayesian analyses.

## Figure S1. Estimates of counterfactual bHIV by the adherence-efficacy method under varying assumptions and corresponding efficacy estimates and quantitative bias analysis


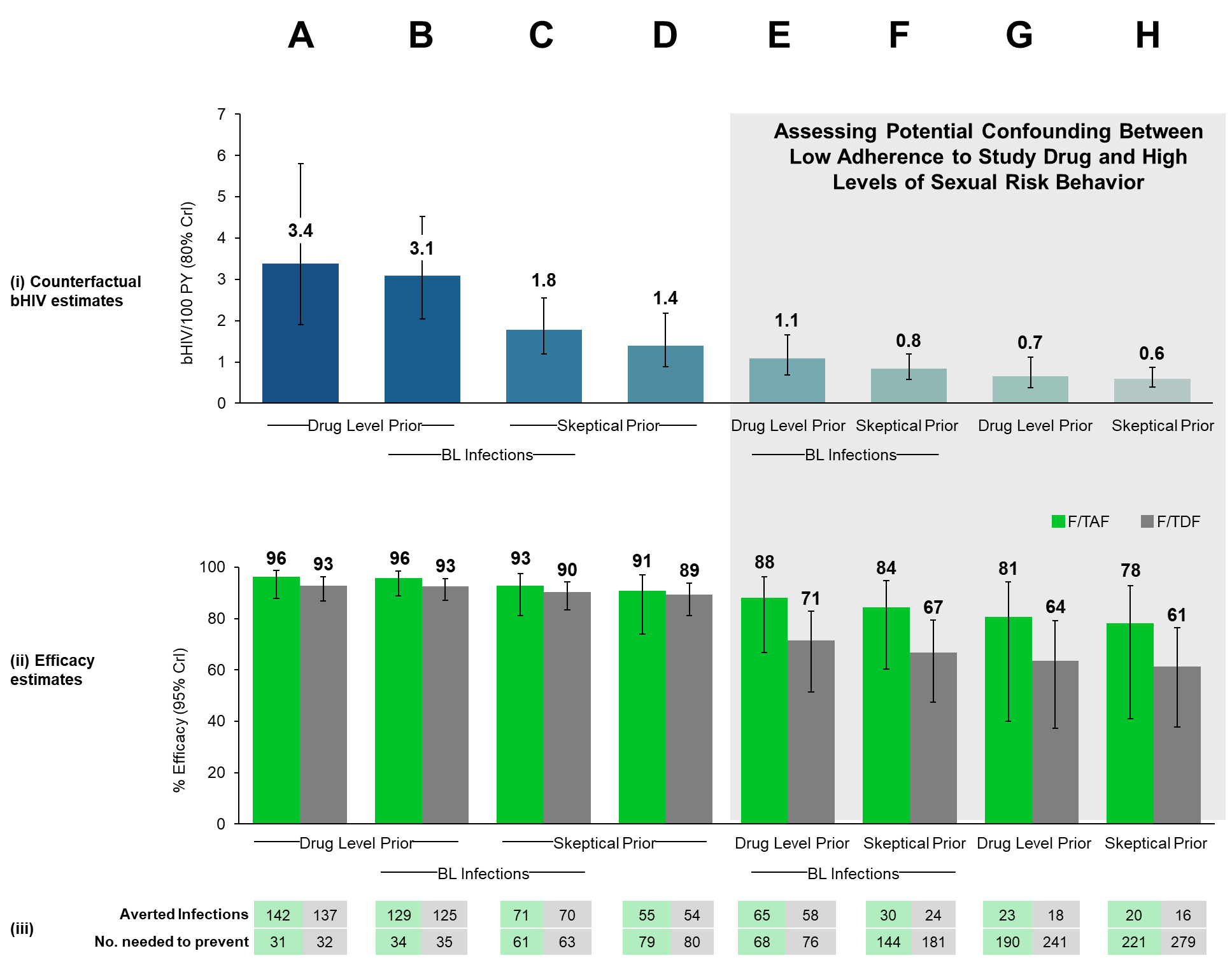


bHIV, background HIV incidence; BL, baseline.
